# A systematic review of the surgical anatomy of the orbital apex

**DOI:** 10.1007/s00276-020-02573-w

**Published:** 2020-10-31

**Authors:** Ӧ. Engin, G. F. J. P. M. Adriaensen, F. W. A. Hoefnagels, P. Saeed

**Affiliations:** 1 Orbital Center, Ophthalmology Department, Amsterdam UMC, location AMC, Amsterdam, The Netherlands; 2 Otorhinolaryngology Department, Amsterdam UMC, location AMC, Amsterdam, The Netherlands; 3 Neurosurgery Department, Amsterdam UMC, location AMC, Amsterdam, The Netherlands

**Keywords:** Orbit, Apex, Anatomy, Surgery

## Abstract

**Purpose:**

The orbital apex is the narrowest part of the orbit, housing the link between the intracranial cavity and orbit. Knowledge of orbital apex anatomy is crucial to selecting a surgical approach and reducing the risk of complications. Our purpose is to summarize current knowledge on surgical anatomy and attempt to reach a consensus on definition of the orbital apex.

**Methods:**

The online databases of Embase, the Cochrane library, Web of Science and PubMed (MEDLINE) were queried in a comprehensive bibliographic search on the (surgical) anatomy of the orbital apex and consisted of a combination of two subjects, using indexed terms and free text: “Orbital Apex” and “Orbital Anatomy.”

**Results:**

A total of 114 relevant papers were included in this review. Numerous anatomical variations are described in the literature. Variations of the optic canal include duplication (0.64%) and keyhole anomaly (2.65%). Variations in pneumatization of the anterior clinoid process were unilateral in almost 10%, bilateral in 9%, and normal in 72%. A rare variant of the superior orbital fissure (SOF) is Warwick’s foramen, which appears as if the lowest portion of the SOF was separated from the main fissure by a transverse bony bridge.

**Conclusion:**

The definition of the orbital apex varies in the literature, and further research would most likely identify additional variations. A universal definition reporting these variations and pathology and imaging findings is essential for determining the optimal surgical approach to the orbital apex.

**Electronic supplementary material:**

The online version of this article (10.1007/s00276-020-02573-w) contains supplementary material, which is available to authorized users.

## Introduction

The orbital apex is the area between the orbit and intracranial space that houses structures like the optic canal (OC), superior orbital fissure (SOF), and inferior orbital fissure (IOF), forming an opening to the orbit [2, 19, 30, 52, 92, 94, 108]. The superolateral orbit can be divided in the frontal one-third to the lacrimal fossa part and the posterior two-thirds connecting the SOF [55]. The part between the posterior ethmoidal foramen and the openings of the OC and SOF has been described as the orbital apex [48].

Detailed anatomical knowledge of the orbital apex is essential for diagnostic purposes and surgical interventions because critical structures are only millimeters apart [27, 79, 92, 104]. The literature describes many variations of the anatomy orbital apex and its structures. There is no precise definition of what the orbital apex comprises and its precise location. Here, we systematically review all current data and reports on the anatomy of the orbital apex. An attempt was made to develop a conclusive definition of the orbital apex.

## Methods

We performed a systematic literature search on the (surgical) anatomy of the orbital apex on June 27, 2017, using online databases including Embase, the Cochrane library, Web of Science, and PubMed (MEDLINE). Preferred Reporting Items for Systematic Reviews and Meta-Analyses (PRISMA) guidelines were followed to query these online databases. The search strategy was developed by an experienced clinical librarian and consisted of a combination of two subjects using indexed terms and free text: “Orbital Apex” and “Orbital Anatomy.” All studies describing data on anatomy alone or surgery techniques focusing on the anatomy were included. Studies focusing on animals, syndromes, fractures and other orbital abnormalities, non-English studies, and reviews were excluded from the analysis. Two reviewers (ÖE and PS) independently assessed all titles, abstracts and the reference lists for relevance. Subsequently, a full-text analysis was performed on all relevant publications for final inclusion in the analysis. Discrepancies regarding the inclusion of eligible studies were mutually resolved by discussion between the two reviewers. The search strategy is included in the Appendix.

## Results

**Fig. 1:**
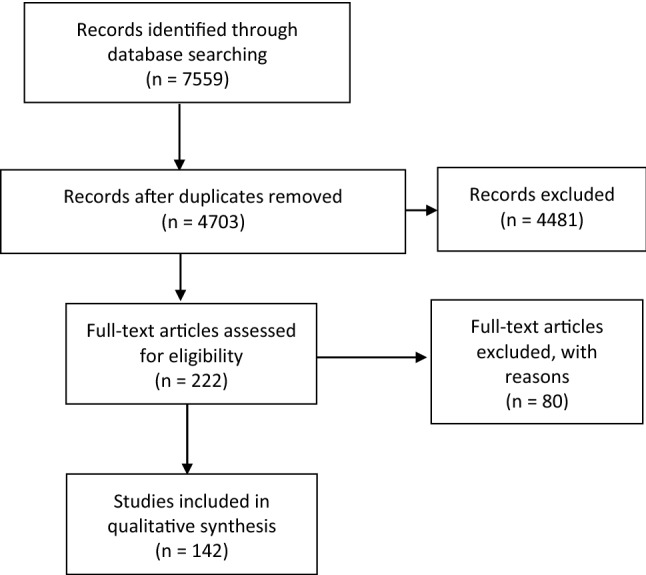
Flow chart search

Our search strategy yielded 4703 papers after removal of duplicates (Table 1). The full texts of 222 articles describing the anatomy of the orbital apex and the important structures related to this area were assessed for eligibility. This resulted in the inclusion of 114 relevant papers in this review (Fig. 1).

**Table 1:** Database results

Database	Papers
Embase.com	3342
Medline Ovid	943
Web of science	283
Cochrane CENTRAL	16
Google scholar	119
Total	4703

### Orbital apex

**Fig. 2:**
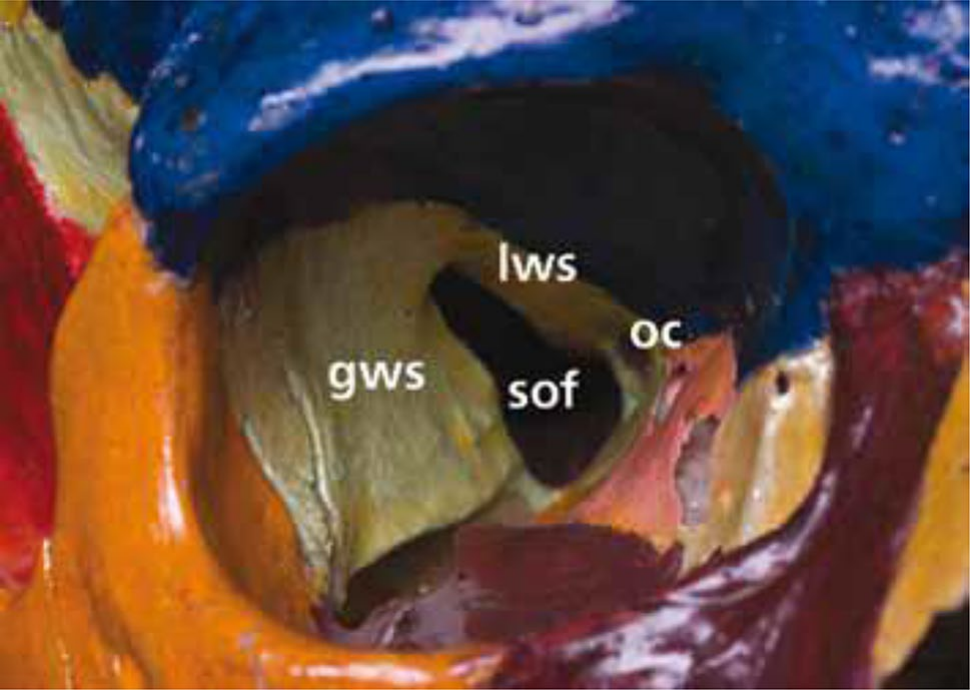
The optic canal (OC) is bordered by the sphenoid bone, superior by the lesser wing, inferolateral by the optic strut and medial by the body. The SOF is localized inferolateral to the optic canal separated by the optic strut and bordered by the lesser wing of the sphenoid superiorly and medially, by greater wing of the sphenoid bone laterally and the orbital process of the palatine bone inferiorly. *GWS* greater wing of the sfenoid, *LWS* lesser wing of the sfenoid, *OC* optic canal, *SOF* superior orbital fissure

The bony orbital apex is the narrowest part of the orbit. The lesser wing of the sphenoid bone forms the roof of the apex, the ethmoidal sinus forms the medial wall, the greater wing of the sphenoid forms the lateral wall, and the orbital plate of the palatine bone forms the floor [1, 30, 46, 63, 82]. The OC is bordered by the sphenoid bone, superior by the lesser wing, inferolateral by the optic strut, and medial by the body. The SOF is inferolateral to the OC separated by the optic strut and bordered by the lesser wing of the sphenoid superiorly and medially, by the greater wing of the sphenoid bone laterally and the orbital process of the palatine bone inferiorly (Fig. 2) [27].

Various studies have measured the distance to the orbital apex. Danko and Haug reported that the distance from the infraorbital rim to the orbital apex (annulus of Zinn) was 39.1 mm (range 33.6–41.6 mm) [36]. Other studies measured the distance from the rim to the optic foramen between 47.93–49.60 mm inferiorly, 41.32–46.43 mm medially from the lacrimal crest, and 44 mm from the lateral orbital rim to the apex [61, 64, 105]. Yilmazlar et al. measured the distance between the orbital apex (described as the part where the OC terminates and the orbit begins) to the tubercular recess as 11.22–11.47 mm [111]. René reported that the orbital apex is located 44–50 mm posteriorly [92]. Smerdon stated that the distance between the back of the eye and the apex is slightly less than the length of the intraorbital part of the optic nerve (ON) [102].

### Optic canal

**Fig. 3:**
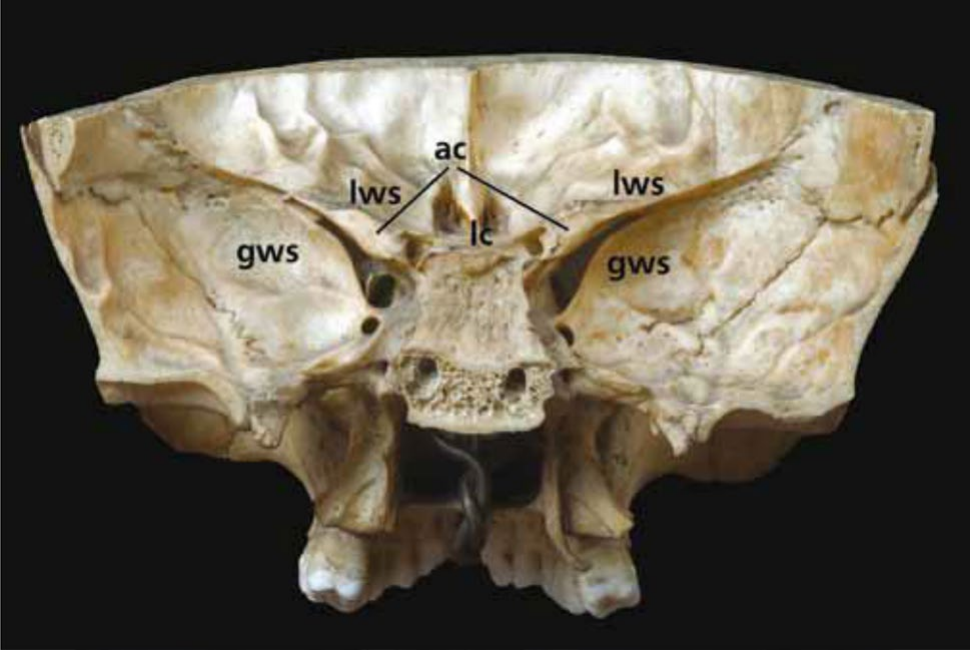
ACP is by far the most important bony projection which hides part of the ON and The ACP is a small triangle bone formed by the medial and posterior end of the lesser wing of the sphenoidale bone. The posterior root of the sphenoidal bone extends medially from the ACP and forms the roof of the OC. *AC* anterior clinoid process, *GWS* greater wing of the sfenoid, *LWS* lesser wing of the sfenoid, *LC* lacrimal crest

The OC contains the ON, ophthalmic artery (OA), and the postganglionic sympathetic nerves that arise from the carotid plexus [1, 21, 22, 30, 35, 46, 50, 52, 63, 79, 88, 90, 95, 97, 101, 105]. The ON is medial to the OA. The OC with its intraorbital end (optic foramen), is bordered medially by the body of the sphenoid bone, superiorly by the superior root of the lesser wing of the sphenoid bone, inferolaterally by the optic strut (the posterior root of the lesser wing of the sphenoid bone), and laterally by the anterior clinoid process (ACP) (Figs. 2 and 3) [8, 14, 21, 27, 30, 34, 46, 50–52, 63, 88, 91, 92].

Variations of the OC include duplication (0.64–2.98% of orbits), which is often associated with the internal carotid artery (ICA) originating from the cavernous sinus. When duplication is present, the lower canal contains the OA and the higher canal contains the ON (Table 2) [15, 16, 28, 48, 66, 81, 98]. Another variation is the keyhole anomaly (1.64–2.65% of orbits), in which the OC has a grooved floor, previously known as a grooved OC. On radiographs, the OC appears in the shape of a keyhole (Table 2) [15, 16, 66, 67].

**Table 2:** OC, ACP and OA variations

Study	*N*	OC duplication	Keyhole anomaly
Bertelli [15]	943	1.96%	2.65%
Ghai et al. [48]	194	2.57%	
Keyes [66]	2187	0.11%	1.64%
Singh [98]	435	2.98%	
Magden et al. [81]	369	0.54%	
Kier [67]	1000		2.20%

ACP variations of the ACP					
Study	*N*	Pneumatization	Bony fusion of the ACP and PCP	Complete bony fusion of the ACP and MCP	Incomplete bony fusion of the ACP and MCP
Mikami et al. [84]	300	14.7%			
Avci et al. [7]	7	28%			
Dagtekin et al. [32]	15		5%	15%	10%
Kim et al. [68]	35		6%	13%	24%

Abnormal origin of the OA								
Study	*N*	CS	ICA	Choroidal segment of the C4 tract of the ICA	ACA	MMA	PCA	BA
Matsumura et al. [83]	109	0.42–14.1%	26.6					
Horiuchi et al. [59]	156		6.7					
Indo et al. [60]	855		3%	0.35%	0.23%			
Hayreh et al. [56]	170	7.50%	4.70%		0.58%	2.40%	0.58%	0.58%
Uchino et al. [106]	826					2.40%		
Bertelli [16]		3.3–8%						

*ACA* anterior cerebral artery; *ACP* anterior clinoid process; *BA* basilar artery; *CS* cavernous sinus; *ICA* internal carotid artery; *MCP* middle clinoid process; *MMA* middle meningeal artery; *OA* ophthalmic artery; *OC* optic canal; *PCA* posterior communicating artery; posterior clinoid process

### Optic nerve

The ON can be divided into four segments: intraocular, intraorbital, intracanalicular, and intracranial [5, 12, 18, 24, 30, 38, 45, 51, 70]. The intraorbital part is located within the intraconal space of the orbit and extends from the globe to the orbital apex, traveling from the globe inferomedially and then superiorly to the optic foramen [12, 45, 46]. The intracanalicular part is located above the OA as it passes through the OC and lesser wing of the sphenoid bone, surrounded by a muscular cone [5, 12, 45, 46]. The intracanalicular and intraorbital parts of the ON are covered by the pia, arachnoid, and dura [45, 101]. The intracranial ON segment passes from the OC anterolaterally to the posteromedial optic chiasm. The intracranial portion of the ON runs along the medial aspect of the ACP before the ON turns toward the optic chiasm [10, 12, 45, 46, 101].

### Anterior clinoid process

Among the bony structures around OC, the ACP is by far the most important bony projection that contains parts of the ON and OA. The ACP is a small triangular bone formed by the medial and posterior end of the lesser wing of the sphenoid bone. The posterior root of the sphenoidal bone extends medially from the ACP and forms the roof of the OC (Fig. 3) [10, 32, 77]. The ACP is composed of a thin shell of outer cortical bone surrounding inner spongy bone [4, 32]. Pneumatization of the ACP was found in 14.7–28% of cases, and a normal ACP was found in 72% of scans (Table 2) [7, 84]. Several studies found bony fusions between the ACP and posterior clinoid process (PCP) and between the ACP and middle clinoid process (MCP). There was a fusion between the ACP and PCP in 5–6% of cases. Incomplete and complete ACP-MCP fusion were seen in 10–24 and 13–15% of cases, respectively (Table 2) [32, 68].

Dural folds cover the ACP, with two dural rings near the ACP and clinoidal ICA. The distal dural ring is formed by the dura from the superolateral part of the ACP, while the proximal one is formed by dura from the inferomedial part of the ACP. These dural rings are an important landmark for the termination of the cavernous segment of the ICA and the beginning of the clinoidal segment, and the distal ring marks the termination of the clinoidal segment of the ICA and the beginning of its ophthalmic segment [71, 76, 96].

ACP removal is possible for non-vascular lesions, such as sphenoid ridge meningiomas and suprasellar lesions, including pituitary adenomas and craniopharyngiomas [10, 37, 39, 58, 71, 86, 112]. It usually should be removed by unroofing of the OC as in spheno-orbital meningiomas to provide exposure, mobilization of the ON, and resection of the tumor extending into the OC. The ACP is closely related to the clinoidal segment of the ICA and the intracranial part of the ON, so its removal carried a risk of potential injury to the clinoidal and ophthalmic segments of the ICA and ON [3, 26, 32, 69].

### Annulus of Zinn and extraocular muscles

In 1780, Zinn described the “tendinem verum,” a fibrous cord below and external to the optic foramen. This cord is divided in three parts: a middle band attached to the inferior rectus, an inner band attached to the medial rectus, and an external band attached to the lateral rectus [8, 35, 79]. The annulus of Zinn contains the ON, OA, oculomotor nerve, abducens nerve, trochlear nerve, nasociliary nerve, the sympathetic roots of the cervical ganglion, and the superior ophthalmic vein (SOV) [19, 21, 27, 50, 63, 95]. The abducens and oculomotor nerves both innervate the extraocular muscles from within the intraconal space after passing through the annulus of Zinn, while the trochlear nerve innervates the superior oblique muscle extraconal. The inferior ophthalmic vein (IOV) passes below the annulus [95, 107].

The annulus of Zinn also surrounds the optic foramen, oculomotor foramen, and the inferior portion of the SOF, creating intraconal and extraconal spaces in the SOF [19, 46, 50, 52, 63, 91, 92]. The opening called the optic foramen (Zinn’s ring) is the bulging end of the SOF and contains the nasociliary, oculomotor, abducens, fascial, and trigeminal nerves, as well as the sympathetic root of the ciliary ganglion [52, 79]. Except for the inferior and superior oblique muscles, all four extraocular muscles originate at the orbital apex from the annulus of Zinn and form a cone that separates extraconal and intraconal spaces [8, 19, 21, 22, 34, 50, 63, 79, 82, 92, 95, 102, 107]. The inferior oblique muscle originates from the periosteum of the maxilla [22, 102]. It is composed of two tendinous portions: inferior and superior. The lower tendon is located inferior to the optic foramen and serves as an origin for the lateral, inferior and medial rectus muscles. The tendon of Lockwood (upper tendon) serves as origin for the superior rectus muscle [30, 46].

The origins of the rectus muscles are separated from each other by thin connective tissue septa. The superolateral intermuscular septum was as described by Ettl et al. in 1997 and 1998 [44, 45]. Koornneef described thin radial septa, which coursed from the rectus muscle to the orbital walls. In two studies, he reported connective tissue strands between both oblique muscles and Tenon’s capsule [72, 73]. More posteriorly, he described septa between the inferior oblique muscle and the periorbit of the orbital floor. Connections were seen between the upper end of the medial rectus muscle and the superior oblique muscle. Multiple connections are described between the lateral and inferior recti muscles and the Müller muscle with three connective tissue septa, and two septa also connected with branches of the inferior ophthalmic vein. Firm attachments of the lateral rectus muscle to the lateral wall were seen. Other branches of the IOV were observed in the septa between the lateral and inferior recti. The lateral rectus muscle had connections with the SOV hammock and ON. Radial septa originate medially from the medial rectus muscle, connecting it with the ethmoidal periorbit. Superiorly to this muscle, septa connect with the orbital roof, the superior oblique muscle, and SOV [72, 73].

### The superior orbital fissure and inferior orbital fissure

The SOF is inferolateral to the OC, between the greater and lesser wings of the sphenoid bone, with a round part inferiorly and a thinner part superolaterally. The SOF can be racket-shaped or round, with a narrow part superotemporally and a wider part medially, below the optic foramen. It separates the posterior segment of the lateral orbital wall from the roof. The SOF connects the middle cranial fossa to the orbit and is separated from the OC and foramen rotundum by the optic and maxillary struts, respectively (Fig. 3) [8, 18, 19, 21, 22, 27, 30, 33, 34, 46, 49, 50, 63, 90–92, 95, 101, 105].

The annulus of Zinn divides the SOF into three parts. The superolateral part contains the frontal, lacrimal and trochlear nerves. The nasociliary nerve, superior and inferior branches of the oculomotor nerve, and the abducens nerve are in the central part; also, the SOV passes through the central part and continues to the cavernous sinus. The inferior part does not have any structures [19, 22, 27, 35, 45, 46, 49, 50, 63, 79, 90, 91, 93, 95]. The OA enters the orbit through the SOF in 5–6% of cases as a result of an aberrant development of arterial blood supply to the orbit [90].

A rare variant of the SOF is Warwick’s foramen, which in many cases is located between the inferior end of the SOF and the foramen rotundum. It separates the SOF from the main fissure by a transverse bony bridge and connects the middle cranial fossa with the orbit [15, 90, 109]. Warwick’s foramen must not be confused with the nearby foramen rotundum itself, which is located further down [90, 109]. Bertelli and Regoli described the presence of this structure in 0.61–0.74% of the cases, unilateral in all investigated skulls and in patients between 14–90 years of age. Their studies described variations in shape as round or crescent foramen, and variations in size where the caliber of the rounded foramina ranged 0.5–2 mm, and the crescentic foramina had a diameter as large as 2.88 mm (SD ± 0.956 mm). It is never found in fetal skulls [15, 90, 109]. Warwick hypothesized that this foramen might contain the IOV, which was also seen by Bertelli and Regoli. They described that the foramen points towards the pterygopalatine fossa; this makes it possible that a vessel is present connecting the cavernous sinus and the pterygoid venous plexus [15, 90, 109].

**Fig. 4:**
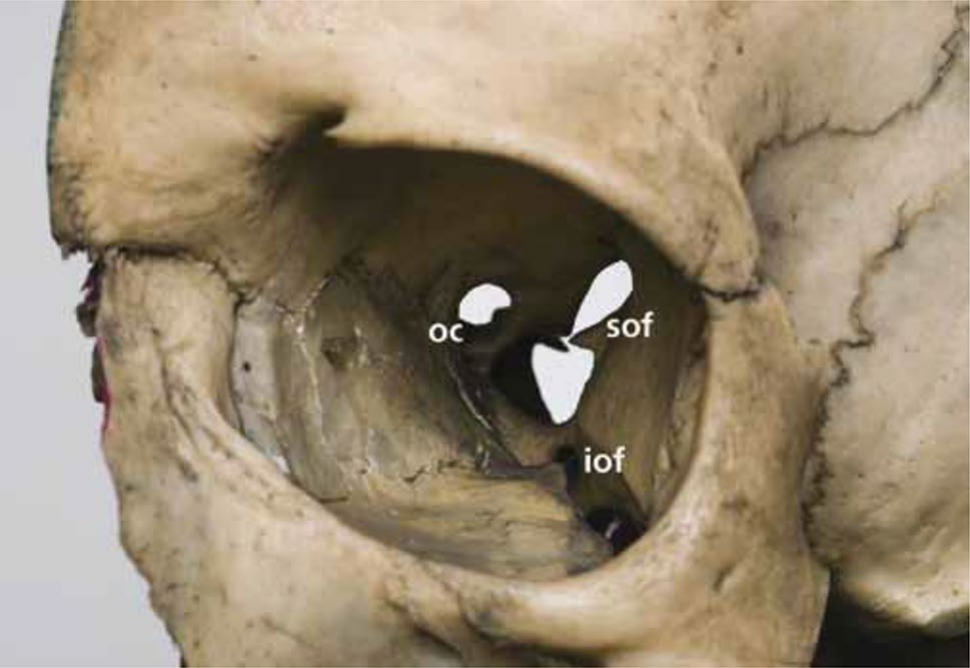
The optic canal and it’s relation to the superior and inferior fissures. *OC* optic canal, *SOF* superior orbital fissure, *IOF* inferior orbital fissure

The IOF is located between the lateral wall and floor of the orbit. The zygomatic bone forms the anterior margin, the sphenoid bone forms the lateral margin, and the maxillary bone forms the medial margin. The anterior part of this fissure is round and the posterior part is thin and obliquely oriented. Anteriorly, the channel is bordered laterally and medially by the sphenoid and maxillary bones, respectively. It connects inferolaterally to the infratemporal fossa [8, 19, 34, 46]. The IOF contains the infraorbital artery, infraorbital nerve, maxillary division of the trigeminal nerve, zygomatic nerve, and branches of the IOV (Fig. 4) [19, 35, 46, 79, 95].

The main arterial supply to the orbit and ON is provided by the OA via the supraclinoid portion of the ICA [22, 46, 55, 56, 70, 78, 87, 91, 92, 102]. In the intracranial segment, the OA is related to the proximal part of the ICA. It makes its way through the subarachnoid space into the intracanalicular segment below the ON and then continues into the OC, mostly inferolateral to the ON to perforate the sheath at the OC exit [16, 18, 19, 21, 30, 40, 42, 45, 46, 50, 52, 55, 56, 62, 63, 70, 89, 91, 92, 101, 102, 113]. OA entrance into the orbit through the SOF is only described in 5–6% of cases due to an aberrant development of the arterial blood supply [90]. Lang et al. reported that in 2–4% of cases, the OA is located in a separate bony canal parallel to the OC [74, 75].

The OA courses below the superior rectus muscle, eventually reaching the medial orbital wall. The main trunk travels near the medial wall of the orbit and splits into terminal branches to supply structures within the orbit [52, 57, 62, 74, 89, 92, 101]. After coursing nasally and anteriorly, the OA runs superior to the ON where it gives off most of its major branches, including the central retinal artery (CRA), which passes inferiorly to the ON, pial vessels, and the posterior ciliary arteries [40, 42, 46, 52, 53, 92]. In 40 and 20% of cases, the CRA originated from the posterior long ciliary artery and posterior ciliary artery, respectively [52, 87]. The CRA penetrates the dural sheath to reach the ON ~ 1 cm behind the globe, to reserve a central position within the ON [40, 46, 87, 92]. After rising from the ON within the globe, it branches into the arteria nasalis retinae superior and arteria nasalis retinae inferior [40]. Small pial vessels branch from the intraorbital part of the OA, the recurrent branch of the posterior ciliary artery, and branches of the CRA and penetrate the superior surface of the optic sheath at a right angle to form a subpial meshwork. This network contributes to the vascularization of the ON fibers [41, 43, 103].

In the literature, an abnormal origin of the OA is described in 1.89–3.1% of cases [13]. Various abnormal origins are described, such as within the cavernous sinus, due to a persistently dorsal OA (0.42–14.1%), C3 tract of the ICA (3–26.6%), or choroidal segment of the C4 tract of the ICA (0.18–0.35%). The origin can be as double arteries with an additional caudal artery passing through the SOF from the ICA bifurcation, middle cerebral artery with unilateral or bilateral ICA absence, anterior cerebral artery (0.23%), posterior communicating artery (0.58%), basilar artery (0.58%), or middle meningeal artery (1.2–4%) (Table 2) [13, 16, 56, 59, 60, 83, 106]. Studies also described variations with contributions from the maxillary and middle meningeal arteries branching from the external carotid artery, where the OA does not arise from the ICA but branches from the middle meningeal artery and enters the orbit through the SOF [16, 55, 56, 78]. The OA can have two supplying branches: a large branch from the middle meningeal artery and a smaller branch from the ICA. A persistent connection is found between the middle meningeal artery and OA as the recurrent meningeal branch of the lacrimal artery. The OA has also been found to arise extradurally from the clinoidal segment of the intracavernous portion of the ICA, where it passes through the SOF instead of the optic foramen [16, 55, 56, 80, 82, 110].

First described by Hayreh et al. in 1963, the blood supply of the ON remains controversial to this day. The discussions revolve around the role of the CRA, the presence of a separate CRA for the ON itself, and the presence of anastomoses between the CRA and arteries surrounding the ON [54, 57]. In 1954, Francois and Neetens suggested that the CRA was strictly terminal without branches throughout its course and proposed that if it had branches, it would supply the retina and papilla as opposed to the ON itself.[47, 54]. In 1960, Singh and Dass described three branches of the CRA in 82% of cases that supply the retina. They did not find intraneural branches that supply the retina as suggested by Francois and Neetens in 1954 [47, 99]. The intraorbital branches supply the dural sheath. The intravaginal branches are the most prominent and are similar to the intraneural branches. The intraneural branches form the axial vascular system of the ON and contain the central vessels [47, 99, 100, 103]. Hayreh also described the presence of multiple branches with various origins [53].

There are also controversies about the site of branch origination. Multiple studies described capillary branches from the CRA in the lamina cribrosa [20, 103]. Singh & Dass suggested that the intraorbital portion ascends all along the entire course of the CRA, the intravaginal part arises from the distal half, and the intraneural parts are evenly distributed [100]. They found no branches at the level of the lamina cribrosa, as described elsewhere [103]. On the other hand, Francois & Neetens proposed that the intraneural branches originate in the anterior third of the CRA behind the lamina cribrosa. These branches are the early retinal branches as described by them and do not supply the ON [47]. Others reported that all the intraneural branches supplied the ON [54, 57, 103].

### Venous drainage of the orbital apex

The main venous drainage is provided by the ophthalmic vein [22, 50, 63, 82, 91]. It has a valveless superior and inferior division that join in the orbital apex and exit through the SOF and IOF [8]. The superior root is the continuation of the supraorbital vein, while the inferior root is the terminal part of the angular vein from the facial venous system [25].

The SOV is found superomedially near the trochlea, lateral to the superior oblique muscle, and passes backwards above the ON and under the superior rectus. The superior supraorbital vein and inferior angular join to form the SOV coursing through the orbit from the front toward the back next to the artery and passing under the superior rectus muscle entering the muscle cone. The SOV can be divided in three segments. The first is the union of the two roots in the anterior orbit. The second segment is where the SOV travels under the superior rectus muscle in the mid-orbital region. The third part starts where the SOV proceeds in a posteriorly extending diagonal course from the second segment, crossing from medial to lateral to reach the lateral border of the superior rectus muscle. From there, it travels posteriorly beside the lateral edge of the superior rectus muscle, then passes the annulus of Zinn and SOF to drain into the cavernous sinus [9, 19, 25, 30, 51, 55, 91, 102]. The IOV is found on the orbital floor and courses along the inferior rectus [30, 34, 92]. It originates as a plexus on the floor of the orbit and drains directly—or indirectly via the SOV—into the cavernous sinus [19, 30].

## Discussion

This literature review summarizes the surgical anatomy and variations of the orbital apex described in the literature. This is to our knowledge, the first systematic review describing the anatomical variations in orbital apex structures to help surgeons navigate in this complex anatomical area.

The reviewed literature reported great variation in different aspects of orbital apex anatomy. These variations can possibly be explained by the fact that anatomical descriptions are not always based on healthy individuals. The majority of studies described the anatomy of patients undergoing surgery or imaging. Therefore, it cannot be excluded that these variations might be more common in pathologic cases. Other possible explanations for these variances in description include selection bias, study size, and universal definitions. Anatomical structures can vary between various populations and diverse individuals, yielding different results. So, normal anatomy and variations are difficult to determine. Baretto et al. described significant differences for certain measures of globe and orbital positions between black and white populations [11]. Kato et al. also found differences in the internal orbital and middle facial breadths in Peruvian, Asian, European, and African populations [65]. Cutrich et al. and Aziz described racial differences in supraorbital notch occurrence in white and black population [9, 31]. Others also reported variable locations of the supraorbital notch [6, 29, 85]. Blake et al. summarized all racial and ethnic differences in ocular and orbital anatomy [17]. To date, a racial variation of the apex has not been described.

There are many controversies regarding the definition and location of orbital apex. We found numerous studies investigating the distance of the orbital apex [36, 61, 64, 92, 102, 105, 111]. There was considerable heterogeneity in the measurements between these studies due to the use of different reference points. Most studies measured the orbital apex from the rim to the optic foramen, but several used the annulus of Zinn as a reference point [36, 61, 64, 92, 102, 105, 111]. To create a clear definition of the orbital apex, a fixed reference point must be adopted. For practical reasons, an easily detected structure, such as bone-like reference point, should be used.

OC variations include duplication and the keyhole anomaly. If a duplication is present, the ON courses through the higher canal [15, 16, 28, 48, 66, 67, 81, 98]. The ACP is often removed in case of non-vascular lesions (e.g., sphenoid ridge meningiomas) and suprasellar lesions (e.g., pituitary adenomas and craniopharyngiomas) [10, 37, 39, 58, 71, 86, 112]. This is an important bony projection because it contains parts of the ON and OA, and it should be treated with caution to avoid surgical complications. Noting the appearance of variations, like pneumatization of the ACP, is important to avoid intraoperative complications like cerebrospinal fluid leaks [7, 84]. Additionally ACP–PCP and ACP–MCP fusions have been described, but the clinical implications have not been detailed in the literature [32, 68]. When fusion is present, ACP dissection is more challenging. Normally you can remove the ACP directly from the bony attachment with the optic strut. But in case of a bony bridge, the ACP remains attached superiorly after dissection. Removal of this portion creates a sharp edge that could damage the carotid artery or ON.

We found no variations regarding the annulus of Zinn and extraocular muscles in the literature. The annulus of Zinn divides the SOF into three parts: superolateral, central, and inferior [19, 22, 27, 35, 45, 46, 49, 50, 63, 79, 90, 91, 93, 95]. A rare variant of the SOF is Warwick’s foramen, which separates the SOF from the main fissure by a transverse bony bridge [15, 90, 109]. Warwick’s foramen possibly contains the inferior ophthalmic vein, but no clinical implications were reported in the reviewed literature [15, 90, 109]. No variations of the IOF have been described.

Variations in the arterial supply of the orbit include abnormal origin of the OA, contribution from the maxillary and middle meningeal arteries to the OA, and variations in the role and branching of the CRA [13, 16, 47, 54–57, 59, 60, 78, 80, 82, 83, 100, 103, 106, 110]. Anastomosis networks exist between branches of the external carotid artery and these OA branches [54, 57]. CRA obstruction will lead to blindness, but OA obstructions may be asymptomatic due to collateral filling from the external carotid artery [23, 70].

## Conclusion

The orbital apex is surrounded by the greater and lesser wings of the sphenoid bone, ethmoidal sinus, and palatine bone, and the annulus of Zinn. However, the precise definition varies in the literature. The degree of anatomical variations in this complex area is immense. Further research would most likely lead to the discovery of more. A universal definition including these variations and pathology and imaging findings is essential for selecting the best surgical approach to the orbital apex. This information will make it possible to predict surgical outcomes for individual patients.

## Electronic supplementary material

Below is the link to the electronic supplementary material.

Supplementary file1 (DOCX 102 kb)

## References

[1] Abed SF, Shams P, Shen S, Adds PJ, Uddin JM (2011). A cadaveric study of the morphometric and geometric relationships of the orbital apex. Orbit.

[2] Abuzayed B, Tanriover N, Gazioglu N, Eraslan BS, Akar Z (2009). Endoscopic endonasal approach to the orbital apex and medial orbital wall: anatomic study and clinical applications. J Craniofac Surg.

[3] Al-Mefty O (1998). Operative atlas of meningiomas.

[4] Cheverud J (1982). Phenotypic, genetic, and environmental morphological integration in the cranium. Evolution.

[5] Anusha B, Harvinder ABRPS (2015). Anatomical variants of surgically important landmarks in the sphenoid sinus: a radiologic study in Southeast Asian patients. Surg Radiol Anat.

[6] Apinhasmit W, Chompoopong S, Methathrathip D, Sansuk R, Phetphunphiphat W (2006). Supraorbital Notch/Foramen, Infraorbital Foramen and Mental Foramen in Thais: anthropometric measurements and surgical relevance. J Med Assoc Thai.

[7] Avci E, Bademci G, Ozturk A (2005). Microsurgical landmarks for safe removal of anterior clinoid process. Minim Invasive Neurosurg.

[8] Aviv RI, Casselman J (2005). Orbital imaging: part 1. Normal anatomy. Clin Radiol.

[9] Aziz SR, Marchena JM, Puran A (2000). Anatomic characteristics of the infraorbital foramen: a cadaver study. J Oral Maxillofac Surg.

[10] Baidya NB, Tang CT, Ammirati M (2013). Intradural endoscope-assisted anterior clinoidectomy: a cadaveric study. Clin Neurol Neurosurg.

[11] Barretto RL, Mathog RH (1999). Orbital measurement in black and white populations. Laryngoscope.

[12] Becker M, Masterson K, Delavelle J, Viallon M, Vargas MI, Becker CD (2010). Imaging of the optic nerve. Eur J Radiol.

[13] Belotti F, Ferrari M, Doglietto F (2016). Ophthalmic artery originating from the anterior cerebral artery: anatomo-radiological study, histological analysis, and literature review. Neurosurg Rev.

[14] Berhouma M, Sc M, Jacquesson T, Sc M, Abouaf L, Sc M, Vighetto A, Ph D, Jouanneau E (2014). Endoscopic endonasal optic nerve and orbital apex decompression for nontraumatic optic neuropathy: surgical nuances and review of the literature. Neurosurg Focus.

[15] Bertelli E (2014). Metoptic canal, duplication of the optic canal and Warwick’s foramen in human orbits. Anat Sci Int.

[16] Bertelli E (2017). An update on the variations of the orbital blood supply and hemodynamic. Surg Radiol Anat.

[17] Blake CR, Lai WW, Edward DP (2003). Racial and ethnic differences in ocular anatomy. Int Ophthalmol Clin.

[18] Bleier BS, Healy DY, Chhabra N, Freitag S (2014). Compartmental endoscopic surgical anatomy of the medial intraconal orbital space. Int Forum Allergy Rhinol.

[19] Braffman BH, Naidich TP, Chaneles M (1997). Imaging anatomy of the normal orbit. Semin Ultrasound CT MRI.

[20] Bron AJ, Tripathi RC, Tripathi BJ, Wolff E (1997). Wolff's anatomy of the eye and orbit.

[21] Burkat CN, Lemke BN (2005). Anatomy of the orbit and its related structures. Otolaryngol Clin North Am.

[22] Chastain JB, Sindwani R (2006). Anatomy of the orbit, lacrimal apparatus, and lateral nasal wall. Otolaryngol Clin North Am.

[23] Chen P, Dunn IF, Aglio LS, Day AL, Frerichs KU, Friedlander RM (2005). Intraoperative awakening for vision examination during ophthalmic artery aneurysm clipping: technical case report. Neurosurgery.

[24] Chen CC, Huang F, Shao HX, Jin JH, Li ZP, Sen ZC (2009). Sectional anatomy of the optic pathways on the coronal plane. J Chinese Med Assoc.

[25] Cheung N, McNab AA (2003). Venous anatomy of the orbit. Investig Opthalmology Vis Sci.

[26] Chi JH, McDermott MW (2003). Tuberculum sellae meningiomas. Neurosurg Focus.

[27] Chong VFH, Fan YF, Chan LL (1999). Radiology of the orbital apex. Australas Radiol.

[28] Choudhry R, Choudhry S, Anand C (1988). Duplication of optic canals in human skulls. J Anat.

[29] Chung MS, Kim HJ, Kang HS, Chung IH (1995). Locational relationship of the supraorbital notch or foramen and infraorbital and mental foramina in Koreans. Acta Anat (Basel).

[30] Cornelius CP, Mayer P, Ehrenfeld M, Metzger MC (2014). The orbits—Anatomical features in view of innovative surgical methods. Facial Plast Surg.

[31] Cutright B, Quillopa N, Schubert W (2003). An anthropometric analysis of the key foramina for maxillofacial surgery. J Oral Maxillofac Surg.

[32] Dagtekin A, Avci E, Uzmansel D, Kurtoglu Z, Kara E, Uluc K, Akture E, Baskaya MK (2014). Microsurgical anatomy and variations of the anterior clinoid process. Turk Neurosurg.

[33] Dallan I, Castelnuovo P, De Notaris M, Sellari-Franceschini S, Lenzi R, Turri-Zanoni M, Battaglia P, Prats-Galino A (2013). Endoscopic endonasal anatomy of superior orbital fissure and orbital apex regions: critical considerations for clinical applications. Eur Arch Oto-Rhino-Laryngology.

[34] Daniels DL, Mark LP, Mafee MF, Massaro B, Hendrix LE, Shaffer KA, Morrissey D, Horner CW (1995). Osseous anatomy of the orbital apex. Am J Neuroradiol.

[35] Daniels D, Pech P, Kay M, Pojunas K, Williams A, Haughton V (1985). Orbital apex: correlative anatomic and CT study. Am J Roentgenol.

[36] Danko I, Haug RH (1998). An experimental investigation of the safe distance for internal orbital dissection. J Oral Maxillofac Surg.

[37] DeJesús O, Sekhar LN, Riedel CJ (1999). Clinoid and paraclinoid aneurysms: surgical anatomy, operative techniques, and outcome. Surg Neurol.

[38] DeMoraes CG (2013). Anatomy of the visual pathways. J Glaucoma.

[39] Dolenc VV (1985). A combined epi- and subdural direct approach to carotid-ophthalmic artery aneurysms. J Neurosurg.

[40] Ehrlich R, Harris A, Moss AM (2010) Anatomy and Regulation of the optical nerve blood flow. In: Dartt D (ed) Encycl eye, 1st edn. Academic Press, pp 73–82

[41] Erdogmus S, Govsa F (2006). Topography of the posterior arteries supplying the eye and relations to the optic nerve. Acta Ophthalmol Scand.

[42] Erdogmus S, Govsa F (2007). Arterial vascularization of the extraocular muscles on its importance for orbital approaches. J Craniofac Surg.

[43] Erdogmus S, Govsa F (2008). Anatomic characteristics of the ophthalmic and posterior ciliary arteries. J Neuro-Ophthalmology.

[44] Ettl A, Kramer J, Daxer A, Koornneef L, Patten S (1997). High-resolution magnetic resonance imaging of the normal extraocular musculature. Eye.

[45] Ettl A, Salomonowitz E, Koornneef L, Zonneveld FW (1998). High resolution MR imaging anatomy of the orbit correlation with comparative cryosectional anatomy. Radiol Clin North Am.

[46] Ettl A, Zwrtek K, Daxer A, Salomonowitz E (2000). Anatomy of the orbital apex and cavernous sinus on high-resolution magnetic resonance images. Surv Ophthalmol.

[47] François J, Neetens A (1954). Vascularization of the optic pathway: I. Lamina cribrosa and optic nerve. Br J Ophthalmol.

[48] Ghai R, Sinha P, Rajguru J, Jain S, Khare S, Singla M (2012). Duplication of optic canal in human skulls. J Anat Soc India.

[49] Govsa F, Kayalioglu G, Erturk M, Ozgur T (1999). The superior orbital fissure and its contents. Surg Radiol Anat.

[50] Guthoff RKJ (2006). Essentials in ophthalmology: oculoplastics and orbit.

[51] Hart CK, Theodosopoulos P V, Zimmer LA (2009) Anatomy of the optic canal : a computed tomography study of endoscopic nerve decompression. 118(12):839–844 10.1177/000348940911801203 20112517

[52] Hayek G, Mercier P, Fournier HD (2006). Anatomy of the orbit and its surgical approach. Adv Tech Stand Neurosurg.

[53] Hayreh SS (1962). The ophtalmic artery: III Branches. Br J Ophthalmol.

[54] Hayreh SS (1963). The central artery of the retina. its role in the blood supply of the optic nerve. Br J Ophthalmol.

[55] Hayreh SS (2006) Orbital vascular anatomy. Eye 20(10):1130–1144 10.1038/sj.eye.6702377 17019411

[56] Hayreh SS, Dass R (1962). The ophthalmic artery: I. origin and intra-cranial and intra-canalicular course. Br J Ophthalmol.

[57] Hayreh SS, Dass R (1962). The ophthalmic artery: II. Intra-orbital course. Br J Ophthalmol.

[58] Heros RC, Nelson PB, Ojemann RG, Crowell RM, DeBrun G (1983). Large and giant paraclinoid aneurysms: surgical techniques, complications, and results. Neurosurgery.

[59] Horiuchi T, Tanaka Y, Kusano Y, Yako T, Sasaki T, Hongo K (2009). Relationship between the ophthalmic artery and the dural ring of the internal carotid artery. J Neurosurg.

[60] Indo M, Oya S, Tanaka M, Matsui T (2014). High incidence of ICA anterior wall aneurysms in patients with an anomalous origin of the ophthalmic artery: possible relevance to the pathogenesis of aneurysm formation. J Neurosurg.

[61] Ji Y, Qian Z, Dong Y, Zhou H, Fan X (2010). Quantitative morphometry of the orbit in Chinese adults based on a three-dimensional reconstruction method. J Anat.

[62] Jo-Osvatic A, Basic N, Basic V, Jukic T, Nikolic V, Stimac D (1999). Topoanatomic relations of the ophthalmic artery viewed in four horizontal layers. Surg Radiol Anat.

[63] Jordan D, Mawn L, Anderson RL (2012) Surgical Anatomy of the Ocular Adnexa: A Clinical Approach (American Academy of Ophthalmology Monograph Series). Oxford University Press

[64] Kang H, Han ÃJJ, Oh H, Kook M, Jung S, Park H (2016). Anatomical studies of the orbital cavity using three-dimensional computed tomography. J Craniofac Surg.

[65] Kato K, Ogata T, Vidal H, Manabe Y, Kitagawa Y, Oyamada J, Rokutanda A (1997). The internal orbital facial breadth and middle facial breadth in Mongoloid crania from Peru and east Asia, with special reference to their significance as a racial criterion. Okajimas Folia Anat Jpn.

[66] Keyes JEL (1935). Observations on four thousand optic foramina in human skulls of known origin. Arch Ophthalmol.

[67] Kier EL (1966). Embryology of the normal optic canal and its anomalies an anatomic and roentgenographic study. Invest Radiol.

[68] Kim JM, Romano A, Sanan A, van Loveren HR, Keller JT (2000). Microsurgical anatomic features and nomenclature of the paraclinoid region. Neurosurgery.

[69] Kinjo T, Al-Mefty O, Ciric I (1995). Diaphragma sellae meningiomas. Neurosurgery.

[70] Kocabiyik N, Yazar F, Ozan H (2009). The intraorbital course of ophthalmic artery and its relationship with the optic nerve. Neuroanatomy.

[71] Komatsu F, Komatsu M, Inoue T, Tschabitscher M (2011). Endoscopic extradural anterior clinoidectomy via supraorbital keyhole: a cadaveric study. Neurosurgery.

[72] Koornneef L (1977). New insights in the human orbital connective tissue. Arch Ophthalmol.

[73] Koornneef L (1988). Eyelid and orbital fascial attachments and their clinical significance. Eye (Lond).

[74] Lang J (1983). Clinical anatomy of the head.

[75] Lang J, Kageyama I (1990). clinical anatomy of the blood spaces and blood vessels surrounding the siphon of the internal carotid artery. Cells Tissues Organs.

[76] Lawton MT (2010) Seven aneurysms: tenets and techniques for clipping. Thieme Medical Publishers Inc

[77] Lehmberg J, Krieg SM, Meyer B (2014). Anterior clinoidectomy. Acta Neurochir (Wien).

[78] Liu Q, Rhoton AL (2001). Middle meningeal origin of the ophthalmic artery. Neurosurgery.

[79] Lockwood CB (1885). The anatomy of the muscles, ligaments, and fasciae of the orbit, including an account of the capsule of tenon, the check ligaments of the recti, and of the suspensory ligament of the eye. J Anat Physiol.

[80] Louw L (2015). Different ophthalmic artery origins: embryology and clinical significance. Clin Anat.

[81] Mağden AO, Kaynak S (1996). Bilateral duplication of the optic canals. Ann Anat.

[82] Martins C, Costa e Silva IE, Campero A, Yasuda A, Aguiar LR, Tatagiba M, Rhoton A (2011). Microsurgical anatomy of the orbit: the rule of seven. Anat Res Int.

[83] Matsumura Y, Nagashima M (1999). Anatomical variations in the origin of the human ophthalmic artery with special reference to the cavernous sinus and surrounding meninges. Cells Tissues Organs.

[84] Mikami T, Minamida Y, Koyanagi I, Baba T, Houkin K (2007). Anatomical variations in pneumatization of the anterior clinoid process. J Neurosurg.

[85] Mwaniki DL, Hassanali J (1992). The position of mandibular and mental foramina in Kenyan African mandibles. East Afr Med J.

[86] Noguchi A, Balasingam V, Shiokawa Y, McMenomey SO, Delashaw JB (2005). Extradural anterior clinoidectomy. J Neurosurg.

[87] Overbeeke JJ, Sekhar LN (2017). Microanatomy of the blood supply to the optic nerve. Orbit.

[88] Purohit BJ (2016). An osteologic study of cranial opening of optic canal in Gujarat region. J Clin Diagnostic Res.

[89] Reeves C, Taylor D (2004). A history of the optic nerve and its diseases. Eye.

[90] Regoli M, Bertelli E (2017). The revised anatomy of the canals connecting the orbit with the cranial cavity. Orbit.

[91] Remington LA (2012). Visual system. Clin Anat Physiol Vis Syst.

[92] René C (2006). Update on orbital anatomy. Eye.

[93] Reymond J, Kwiatkowski J, Wysocki J (2008). Clinical anatomy of the superior orbital fissure and the orbital apex. J Cranio-Maxillofacial Surg.

[94] Sandu K, Monnier P, Pasche P (2012). Anatomical landmarks for transnasal endoscopic skull base surgery. Eur Arch Otorhinolaryngol.

[95] Schiefer U, Wilhelm H, Hart W (2007). Clinical neuro-ophthalmology a practical guide.

[96] Seoane E, Rhoton AL, de Oliveira E (1998). Microsurgical anatomy of the dural collar (carotid collar) and rings around the clinoid segment of the internal carotid artery. Neurosurgery.

[97] Sinanoglu A, Orhan K, Kursun S, Inceoglu B, Oztas B (2016). Evaluation of optic canal and surrounding structures using cone beam computed tomography. J Craniofac Surg.

[98] Singh M (2005). Duplication of optic canal in adult Japanese human skulls. J Anat Soc India.

[99] Singh S, Dass R (1960). The central artery of the retina. I. Origin and course. Br J Ophthalmol.

[100] Singh S, Dass R (1960). The central artery of the retina II. A study of its distribution and anastamoses. Br J Ophthalmol.

[101] Slavin KV, Dujovny M, Soeira G, Ausman JI (1994). Optic canal: microanatomic study. Skull Base Surg.

[102] Smerdon D (2000). Anatomy of the eye and orbit. Curr Anaesth Crit Care.

[103] Steele EJ, Blunt MJ (1956). The blood supply of the optic nerve and chiasma in man. J Anat.

[104] Tepedino MS, Pinheiro-Neto CD, Bezerra TFP, Gardner PA, Snyderman CH, Voegels RL (2016). Endonasal identification of the orbital apex. Laryngoscope.

[105] Turvey TA, Golden BA (2012). Orbital anatomy for the surgeon. Oral Maxillofac Surg Clin North Am.

[106] Uchino A, Saito N, Takahashi M, Kozawa E, Mizukoshi W, Nakajima R, Okano N (2013). Persistent dorsal ophthalmic artery and ophthalmic artery arising from the middle meningeal artery diagnosed by MR angiography at 3 T. Surg Radiol Anat.

[107] Ulutas M, Boyac S, Türker K, Aksoy K (2016). Surgical anatomy of the cavernous sinus, superior orbital fissure, and orbital apex via a lateral orbitotomy approach: a cadaveric anatomical study. Acta Neurochir.

[108] Vohra ST, Escott EJ, Stevens D, Branstetter BF (2011). Categorization and characterization of lesions of the orbital apex. Neuroradiology.

[109] Warwick R (1951). A juvenile skull exhibiting duplication of the optic canals and subdivision of the superior orbital fissure. J Anat.

[110] Wu W, Lu SY, Liu CY, Tu Y, Qian Z (2015). Image-guided endoscopic combined with deep lateral orbitotomy removal of a small foreign body at the deep lateral orbital apex. J Craniofac Surg.

[111] Yilmazlar S, Saraydaroglu O, Korfali E (2012). Anatomical aspects in the transsphenoidal-transethmoidal approach to the optic canal: an anatomic-cadaveric study. J Cranio-Maxillofacial Surg.

[112] Yonekawa Y, Ogata N, Imhof HG, Olivecrona M, Strommer K, Kwak TE, Roth P, Groscurth P (1997). Selective extradural anterior clinoidectomy for supra- and parasellar processes. Technical note. J Neurosurg.

[113] Zoli M, Manzoli L, Bonfatti R (2016). Endoscopic endonasal anatomy of the ophthalmic artery in the optic canal. Acta Neurochir.

