# Supplementary material for: A systematic review of the surgical anatomy of the orbital apex
Source: Surg Radiol Anat. 2020 Oct 31;43(2):169–78. doi: 10.1007/s00276-020-02573-w (PMC7843489; doi:10.1007/s00276-020-02573-w)
Supplement: Supplementary file 1 — Supplementary file1 (DOCX 102 kb) [file 276_2020_2573_MOESM1_ESM.docx]

**Appendix**

**Embase.com 3374**

('optic nerve'/de OR 'extraocular muscle'/exp OR 'oculomotor nerve'/de OR 'ophthalmic vein'/de OR 'ophthalmic artery'/de OR ((orbit* NEAR/3 (apex OR muscle*)) OR ((optic* OR second-cranial) NEAR/3 nerv*) OR (optic* NEAR/3 foramen*) OR (annul* NEAR/3 zinn) OR ((extraocul* OR ocular*) NEAR/3 muscl*) OR ((inferior* OR superior* OR medial OR lateral) NEAR/3 (obliq* OR rectus OR rectal) NEAR/3 musc*) OR (superior NEAR/3 orbital* NEAR/3 (fissure* OR nerve)) OR ((supraorbital* OR oculomotor OR oculo-motor ) NEAR/3 (fissure* OR nerve)) OR (ophthalm* NEAR/3 (vein* OR arter* OR vessel*))):ab,ti) AND ('anatomy'/exp OR 'anatomic model'/de OR 'anatomical variation'/exp OR 'vascularization'/exp OR (anatom* OR vasculari* OR microvasculari* OR vasculature*):ab,ti) OR ((orbit* NEAR/3 apex):ti NOT (orbit* NEAR/6 (syndrome* OR disorder* OR disease* OR fracture* OR tumo* OR inflammat* OR injur* OR lesion* OR wound* OR hemangioma*)):ab,ti) NOT ([animals]/lim NOT [humans]/lim) NOT ([Conference Abstract]/lim OR [Letter]/lim OR [Note]/lim OR [Editorial]/lim) AND [english]/lim

**Medline Ovid 3079**

(optic nerve/ OR Oculomotor Muscles/ OR Ophthalmic Artery/ OR ((orbit* ADJ3 (apex OR muscle*)) OR ((optic* OR second-cranial) ADJ3 nerv*) OR (optic* ADJ3 foramen*) OR (annul* ADJ3 zinn) OR ((extraocul* OR ocular*) ADJ3 muscl*) OR ((inferior* OR superior* OR medial OR lateral) ADJ3 (obliq* OR rectus OR rectal) ADJ3 musc*) OR (superior ADJ3 orbital* ADJ3 (fissure* OR nerve)) OR ((supraorbital* OR oculomotor OR oculo-motor ) ADJ3 (fissure* OR nerve)) OR (ophthalm* ADJ3 (vein* OR arter* OR vessel*))).ab,ti.) AND (exp anatomy/ OR "anatomy and histology".xs. OR Models, Anatomic/ OR (anatom* OR vasculari* OR microvasculari* OR vasculature*).ab,ti.) OR ((orbit* ADJ3 apex).ti. NOT (orbit* ADJ6 (syndrome* OR disorder* OR disease* OR fracture* OR tumo* OR inflammat* OR injur* OR lesion* OR wound* OR hemangioma*)).ab,ti.) NOT (exp animals/ NOT humans/) NOT (letter OR news OR comment OR editorial OR congresses OR abstracts).pt. AND english.la.

**Cochrane CENTRAL 55**

(((orbit* NEAR/3 (apex OR muscle*)) OR ((optic* OR second-cranial) NEAR/3 nerv*) OR (optic* NEAR/3 foramen*) OR (annul* NEAR/3 zinn) OR ((extraocul* OR ocular*) NEAR/3 muscl*) OR ((inferior* OR superior* OR medial OR lateral) NEAR/3 (obliq* OR rectus OR rectal) NEAR/3 musc*) OR (superior NEAR/3 orbital* NEAR/3 (fissure* OR nerve)) OR ((supraorbital* OR oculomotor OR oculo-motor ) NEAR/3 (fissure* OR nerve)) OR (ophthalm* NEAR/3 (vein* OR arter* OR vessel*))):ab,ti) AND ((anatom* OR vasculari* OR microvasculari* OR vasculature*):ab,ti) OR ((orbit* NEAR/3 apex):ti NOT (orbit* NEAR/6 (syndrome* OR disorder* OR disease* OR fracture* OR tumo* OR inflammat* OR injur* OR lesion* OR wound* OR hemangioma*)):ab,ti)

**Web of science 851**

TS=((((orbit* NEAR/2 (apex OR muscle*)) OR ((optic* OR second-cranial) NEAR/2 nerv*) OR (optic* NEAR/2 foramen*) OR (annul* NEAR/2 zinn) OR ((extraocul* OR ocular*) NEAR/2 muscl*) OR ((inferior* OR superior* OR medial OR lateral) NEAR/2 (obliq* OR rectus OR rectal) NEAR/2 musc*) OR (superior NEAR/2 orbital* NEAR/2 (fissure* OR nerve)) OR ((supraorbital* OR oculomotor OR oculo-motor ) NEAR/2 (fissure* OR nerve)) OR (ophthalm* NEAR/2 (vein* OR arter* OR vessel*)))) AND ((anatom* OR vasculari* OR microvasculari* OR vasculature*)) NOT ((animal* OR rat OR rats OR mouse OR mice OR murine OR zebrafish OR monkey* OR primate* OR cat OR cats OR feline OR dog OR dogs OR canine OR cow OR cows OR bovine OR horse OR quine* OR pig OR pigs OR swine OR porcine OR rabbit* OR rodent OR fish) NOT (human* OR patient*))) OR (TI=(orbit* NEAR/2 apex) NOT TS=(orbit* NEAR/5 (syndrome* OR disorder* OR disease* OR fracture* OR tumo* OR inflammat* OR injur* OR lesion* OR wound* OR hemangioma*))) AND DT=(article) AND LA=(English)
